# Associations between internet addiction and psychological problems among adolescents: description and possible explanations

**DOI:** 10.3389/fpsyg.2023.1097331

**Published:** 2023-05-15

**Authors:** Yaqing Xue, Benli Xue, Xiao Zheng, Lei Shi, Pengyan Liang, Shujuan Xiao, Fang Dong, Jiachi Zhang, Yaguang Chen, Yuxi Liu, Zuguo Qin, Chichen Zhang

**Affiliations:** ^1^School of Public Health, Southern Medical University, Guangzhou, Guangdong, China; ^2^School of Health Management, Southern Medical University, Guangzhou, Guangdong, China; ^3^Department of Health Management, Shunde Hospital, Southern Medical University, Guangzhou, China; ^4^Health Publicity and Education Center of Guangdong Province, Guangzhou, Guangdong, China; ^5^Health Education Center of Maoming City, Maoming, Guangdong, China; ^6^School of Humanities and Management, Institute for Health Law and Policy, Guangdong Medical University, Dongguan, Guangdong, China; ^7^Department of Health Management, Nanfang Hospital, Southern Medical University, Guangzhou, China

**Keywords:** adolescents, Internet addiction, psychological problems, interaction, health management

## Abstract

**Introduction:**

Internet addiction (IA) is becoming a significant public health issue that requires urgent attention, especially for adolescents. Previous studies mainly focused on the risk factors of Internet addiction and drawn some conclusions. The purpose of this study is to investigate the Internet addiction status and concurrent psychological problems in adolescent groups, and explore the differences in the relationship between IA and psychological problems in adolescents from gender and grade.

**Methods:**

A cross-sectional study was conducted among students of junior and senior high school in 21 prefecture-level cities of Guangdong Province. The Young Diagnostic Questionnaire (YDQ) and the validated Chinese version of the Depression Anxiety Stress Scale-21 (DASS-21) were used to assess participants’ Internet addiction and psychological status. Two-way ANOVA was used to verify the interaction between gender and Internet addiction, grade and Internet addiction on psychological problems. When the interaction was statistically significant, simple effect analysis was further carried out.

**Results:**

The prevalence of problematic Internet use (PIU), depression, anxiety, and stress symptoms among adolescents were 16.32%, 30.16%, 35.97% and 18.80% respectively. Although the prevalence of PIU among boys (17.89%) was higher than that of girls (14.86%), girls in PIU group had the highest scores of depression, anxiety and stress. Similarly, the interaction between grade and Internet addiction was also significant (*p* < 0.05). Although the prevalence of PIU was the highest in grade 9 (17.29%), the adverse effects of Internet addiction on psychological problems were different in different grades.

**Discussion:**

Internet addiction was prevalent among adolescents in Guangdong province, and psychological problems also cannot be ignored. Thus, this study suggests that long-term follow-ups should be included in mental health programs of adolescents to monitor the development of Internet addiction and psychological problems.

## Introduction

With the rapid popularity of Internet in the 21st century, the Internet economy and knowledge economy have been keeping on working new wonders (Greydanus and Greydanus, 2012). Now we are indeed entering the Information Age. The number of Internet users increases rapidly worldwide given that the Internet altered people’s lifestyles through its advantages in communication, socialization, and even online education (Kitazawa et al., 2018). Recently, the 47th Statistical Survey Report on Internet Development published by China Internet Network Information Center in 2021 showed that the number of Internet users in China has reached 989 million, accounting for one fifth of the world’s Internet users. Significantly, the main body of the growth of Internet users in China is gradually transforming from the youth group to the minors and the elderly group. It is reported that the proportion of young Internet users is 16.6%, and it is increasing year by year. With the improvement of living standards, the current generation of adolescents has grown up with access to computers and the internet from an early age on (Shapiro and Margolin, 2014). In general, adolescents utilize the Internet for various purposes such as entertainment, retrieving information, communication, learning, and others (Straker et al., 2009; Cabinet Office Government of Japan, 2017). Especially during the outbreak of coronavirus disease 2019 (COVID-19), online teaching has become an important way to ensure students’ normal learning. In addition, studies have shown that adolescents play online games or use social media can be helpful in developing and maintaining social relations and friendships (Van den Eijnden et al., 2018). However, excessive internet use has been associated with potentially detrimental side effects, such as Internet addiction.

Internet addiction (IA) can be defined as overuse of the Internet leading to impairment of an individual’s psychological state (both mental and emotional), as well as their scholastic or occupational and social interactions, which is the most common problem among adolescents (Beard and Wolf, 2001; Bisen and Deshpande, 2018). Involving profound individual biological, social, and psychological changes, adolescence is a difficult stage of life in which to change health-related behaviors, with many social interactions being impacted (Garcíacueto, 2014; Pedrosa et al., 2014). As the major source of socialization, internet use plays an integral part in many adolescents’ daily lives (Guan and Subrahmanyam, 2009). However, once addicted to the Internet, it may lead to difficulties in maintaining real-life relationships and damage daily activities (Kubey et al., 2010). One of the most obvious features is the appearance of psychological problems. It is reported that IA was significantly related to mental health problems (Zhao et al., 2023), such as less life satisfaction, mood disorders, anxiety disorders, depression symptoms and so on. Steffen et al. found that the increase of IA may exacerbate pre-existing depression (Steffen et al., 2020). A cross-sectional study in Korean adolescents found that problematic internet use (PIU) was significantly associated with suicidal ideation as well as depression (Park et al., 2013). Salmela-Aro et al. (2017) used longitudinal data to analyze 3,338 adolescents aged 12–18, and showed that among adolescents, excessive internet use can be a cause of school burnout that can later spill over to depressive symptoms. However, are there differences in the relationship between IA and psychological problems among adolescents with different characteristics? Solving this problem has guiding significance for preventing and intervening adolescents’ IA and alleviating psychological problems.

In recent years, scholars have conducted empirical researches on the related factors of IA and have drawn some conclusions. Consistent with other addictive behaviors, many studies have showed that male adolescents are more prone to IA (Shek and Yu, 2016). For example, a study conducted in Europe indicated that the overall prevalence of PIU among adolescents (mean age: 14.9 ± 0.89) was 4.4%, male students (5.2%) had a higher rate of PIU than their female counterparts (3.8%) (Durkee et al., 2012). Tsai et al. (2009) also found that compared with female, male students increased the risk of IA by 1.3-fold (Tsai et al., 2009). Considering the different personalities traits of male and female, the negative impact of PIU on individuals, especially whether there are gender differences in psychological problems, deserves further discussion. In addition, age or grade is also an important determinant. Widyanto and Mcmurran (2004) argued that Internet addiction is “a temporary phenomenon for some individuals, likely related to the initial novelty of the Internet and wearing off with increased familiarity”. Based on six waves of longitudinal data, Shek’s study also found that although there was an initial increase in some Internet addictive behaviors from secondary 1 to secondary 2 (ie, grade 7–8), the proportion of respondents who could be regarded as Internet-addicted gradually decreased throughout the adolescent years (Shek and Yu, 2016). It can be seen that the rate of PIU among adolescents of different ages or grades is different, which may lead to the difference of their psychological problems. So, the associations between IA and psychological problems may vary according to the sex and grade of adolescents.

Building upon previous research in this area, we conducted a cross-sectional study. The purpose of this study is to investigate the phenomenon of IA and concurrent psychological problems in adolescent groups, and explore the differences in the relationship between IA and psychological problems in adolescents from gender and grade, so as to identify any existing associations and theorize as to their causes. We believe that the data from this study will add meaningful information to the field.

## Methods

### Study design and participants

This is a large sample cross-sectional study that approached students of junior and senior high school in 21 prefecture-level cities of Guangdong Province, jointly carried out by Health Promotion and Education Center and Education Department of Guangdong Province. The survey was conducted from May to June 2020, and the target population comprised school-aged students in Guangdong province. To build our sample, a 10% of junior and senior high schools were randomly selected from each city using the equal probability method, then a cluster sampling method was used to extract students from these schools, and the probability of each student being selected was the same. Owing to the impact of the pandemic (COVID-19), the Chinese version of the electronic questionnaire through an online survey platform—Survey-Star (Changsha Ranxing Science and Technology, Shanghai, China) were distributed to adolescents through their teachers, and the recovered copies were reviewed by professionals. The Survey-Star is a website providing online survey and statistical analysis functions (Dong et al., 2020). The questionnaire was anonymous and can only be submitted after all questions are completed to ensure the confidentiality and reliability of data.

The inclusion criteria of school-age students were (1) grades 7–12, boy or girl, (2) voluntary participation in this survey, (3) ability to complete the online survey or with the help of parents. Meanwhile, those who were unwilling to participate in the investigation or were diagnosed with cognitive impairment or serious illnesses were excluded. Finally, 154,705 questionnaires were recovered. A total of 140,782 valid questionnaires were collected after removing invalid questionnaires such as wrong filling, random filling and logic errors, with an effective recovery rate of 91.00%. Ethical approval for this study was obtained from Southern Medical University. A parent or guardian assented to student participation before they completed the survey. Due to the heavy workload of written consents from the respondent, the school staff assisted in informing the respondent of the purpose and procedure of this study.

## Measures

### Internet addiction

The Young Diagnostic Questionnaire (YDQ) was used to assess the status of IA among participants. The 8 items of the YDQ are based on the criteria for pathological gambling in the fourth edition of the Diagnostic and Statistical Manual of Mental Disorders (American Psychiatric Association, 1994), with a “yes” or “no” response and scores ranging from 0 to 8. All participants who answered “yes” to five or more items were categorized as “addictive Internet users” (Young, 2004). The scale has been translated into multiple languages and was used by researchers worldwide (Siomos et al., 2013), Cronbach’s *α* of the scale ranges from 0.68 to 0.79 (Wartberg et al., 2016). In the studied population, the values of Cronbach’s *α* was 0.849, and participants whose YDQ scores ranges from 0 to 4 points were classified as the Normal Internet Use (NIU) group, and the participants whose scores ranges from 5 to 8 points were classified as the Problematic Internet Use (PIU) group.

### Psychological problems

The psychological problem of the respondents was assessed by the validated Chinese version of the Depression Anxiety Stress Scale-21 (DASS-21) in this research. The scale contains 21 items, which was a set of three self-administered subscales (7 questions each) designed to measure the negative emotional states of depression, anxiety, and stress over the prior week (Beiter et al., 2015). Each item assessed symptom severity on a four-point Likert scale scoring from 0 to 3 (0: not at all; 1: some of the time; 2: a good part of the time; and 3: most of the time). The scores of the DASS-21 ranged from 0 to 42, the score of each subscale was summed up and then was multiplied by two according to the guidelines of DASS-21. According to previous studies, the following cut-off scores are used for each subscale: depression: normal 0–9, abnormal 10–42; anxiety: normal 0–7, abnormal 8–42; stress: normal 0–14, abnormal 14–42 (Cheung et al., 2016; Wang et al., 2016). The DASS-21 has been proven to have a good reliability (Henry and Crawford, 2005), the Cronbach’s α of the total scale in this study was 0.961, the Cronbach’s *α* of the depression, anxiety, stress subscales were 0.911, 0.879 and 0.898, respectively.

### Social-demographic and lifestyle information

Sociodemographic characteristics include sex, age, school grade, residence, myopia and boarding. Lifestyle behavior involves sleep quality, exercise time and screen time. Sleep quality was assessed by the following questions: “How do you feel about your sleep quality in the past month?” with a possible answer of “Very good,” “Good,” “Bad,” or “Very bad.” For purposes of analysis, “Very good” and “good” were merged into “Good,” and “Bad,” and “Very bad” were merged into “Bad.” Data on exercise time were obtained by self-reported questionnaires. The participants reported exercise time using the following question: “How many hours per day do spend exercising?” The responses included “within 2 h” and “more than 2 h.” Data on screen time were obtained using the following question: “How many hours per day do you spend on electronic devices (besides study)?” All the time spent on screen-based electronic mediums and devices, such as TV, computer, mobile phone, video games, and tablet TV, were included. The responses were classified into “within 2 h” and “more than 2 h.”

### Statistical analysis

All study variables were analyzed using descriptive analysis. Proportion with percentage was calculated for categorical variables and mean with standard deviation for continuous scale variables. First, Chi-square tests were used to compare the differences of Internet addiction and psychological problems (DASS-21) among adolescents of different genders and ages. Then, we also compared the differences in the depression, anxiety, and stress scores between NIU group and PIU group by two-sample t-test, and included gender and age for further comparison. Next, two-way ANOVA was used to verify the interaction between gender and Internet addiction, grade and Internet addiction on psychological problems. When the interaction was statistically significant, simple effect analysis was further carried out. The level *p* < 0.05 was considered as the cutoff value for significance. All data were analyzed using the statistical software SPSS Version 23.0.

## Results

### Characteristics of study participants

Among the 140,782 participants, 48.26% were boys and 51.74% were girls. The average age was (14.76 ± 1.44), with a range of 11 to 17 years. About half of the them lived in urban areas (51.72%), and 48.28% lived in rural areas. Myopia was found in 82,637 (58.70%) of the participants. And 52.00% of the participants boarded at school, 75.67% had good sleep quality. In total, the mean YDQ score was (2.01 ± 2.36), with 16.32% of adolescent were classified as belonging to the PIU group. And the prevalence of depression, anxiety, and stress symptoms came in at 30.16% (42466), 35.97% (50640) and 18.80% (26,466) according to DASS-21. Others lifestyle information were also presented in Table 1.

**Table 1:** Basic characteristics of the participants (*n* = 140,782).

Variables	*n*	%
Sex		
Boys	67,945	48.26
Girls	72,837	51.74
Age	14.76 ± 1.44	
Grade		
7th	41,229	29.29
8th	34,602	24.58
9th	22,874	16.25
10th	23,067	16.38
11th	144,397	10.23
12th	4,613	3.27
Residence		
Urban	72,813	51.72
Rural	67,969	48.28
Myopia		
Yes	82,637	58.70
No	58,145	41.30
Boarder		
Yes	73,204	52.00
No	67,578	48.00
Sleep quality		
Good	106,527	75.67
Bad	34,255	24.33
Exercise time		
≤2 h	63,613	45.19
>2 h	77,169	54.81
Screen time		
≤2 h	30,034	21.33
>2 h	63,374	78.67
IA		
PIU	22,978	16.32
NIU	117,804	83.68
Depression		
Normal	98,316	69.84
Abnormal	42,466	30.16
Anxiety		
Normal	90,142	64.03
Abnormal	50,640	35.97
Stress		
Normal	114,316	81.20
Abnormal	26,466	18.80

NIU, Normal Internet use (YDQ score ≤ 4 points); PIU, Problematic Internet use (YDQ score ≥ 5 points).

### Comparison of PIU, depression, anxiety, and stress among different sex and grade group

Chi square test found there were significant differences in the prevalence of PIU among different sex (*p* < 0.05). 17.89% of the boys were classified as belonging to the PIU group, which was significantly higher than that of girls (14.86%). Yet, when the grades were divided into junior middle school (7th-9th) and senior high school (10th-12th), there was no significant difference between the two grade groups (16.34% vs. 16.28%, *p > 0.05*). Further analysis showed that there were significant differences in the prevalence of PIU among adolescents in six grades (7th-12th; *p* < 0.05), and the highest prevalence of PIU was among adolescents in 9th-grade (17.29%). In addition, this study also found there were statistical differences in the prevalence of depression, anxiety and stress among adolescents of different sex and grade (*p* < 0.05; Table 2).

**Table 2:** Comparison of PIU, psychological problems among different sex and grade group.

Variables	PIU	Depression	Anxiety	Stress
Sex				
Boys	12,157 (17.89)	20,074 (29.54)	23,093 (33.99)	11,253 (16.56)
Girls	10,821 (14.86)	22,392 (30.74)	27,547 (37.82)	15,213 (20.89)
*p*	<0.001	<0.001	<0.001	<0.001
Grade				
7th-9th	16,127 (16.34)	27,627 (27.99)	32,832 (33.26)	17,265 (17.49)
10th-12th	6,857 (16.28)	14,839 (35.27)	17,808 (42.32)	9,201 (21.87)
*p*	0.793	<0.001	<0.001	<0.001
7th	6,437 (15.61)	10,740 (26.05)	12,881 (31.24)	6,532 (15.84)
8th	5,735 (16.57)	9,815 (28.36)	11,558 (33.40)	6,255 (18.08)
9th	3,955 (17.29)	7,072 (30.92)	8,393 (36.69)	4,478 (19.58)
10th	3,702 (16.05)	7,995 (34.66)	9,538 (41.35)	4,947 (21.45)
11th	2,446 (16.99)	5,186 (36.02)	6,322 (43.91)	3,263 (22.66)
12th	703 (15.24)	1,658 (35.94)	1948 (42.23)	991 (21.48)
*p*	<0.001	<0.001	<0.001	<0.001

PIU: Problematic Internet use (YDQ score ≥ 5 points).

### Association between IA and psychological distress (depression, anxiety, and stress)

Table 3 shows the scores of depression, anxiety and stress among adolescents under different groups of IA. The independent *t*-test showed that the scores of depression, anxiety and stress in PIU group were significantly higher than those in NIU group (*p* < 0.05). Similarly, this difference was also statistically significant between different sex (boys and girls) and grade (junior middle school: 7th-9th, and senior high school: 10th-12th; *p* < 0.05). The results suggested that the mental health problems of adolescents in PIU group were more serious than those in NIU group.

**Table 3:** Scores of psychological problems of adolescents in different IA groups.

Variables	NIU group (*M* ± SD)	PIU group (*M* ± SD)	*p*
Total			
Depression	5.85 ± 7.78	12.37 ± 10.09	<0.001
Anxiety	5.97 ± 7.30	10.91 ± 9.13	<0.001
Stress	7.72 ± 8.21	14.31 ± 9.90	<0.001
Boys			
Depression	5.70 ± 7.85	11.32 ± 9.46	<0.001
Anxiety	5.71 ± 7.42	9.85 ± 8.71	<0.001
Stress	7.22 ± 8.18	12.96 ± 9.43	<0.001
Girls			
Depression	5.99 ± 7.72	13.55 ± 10.63	<0.001
Anxiety	6.21 ± 7.19	12.10 ± 9.44	<0.001
Stress	8.18 ± 8.21	15.83 ± 10.20	<0.001
Grade 7–9			
Depression	5.49 ± 7.66	12.04 ± 10.11	<0.001
Anxiety	5.61 ± 7.18	10.38 ± 9.15	<0.001
Stress	7.29 ± 8.10	13.89 ± 9.87	<0.001
Grade 10–12			
Depression	6.69 ± 7.80	13.15 ± 10.00	<0.001
Anxiety	6.83 ± 7.52	12.16 ± 8.96	<0.001
Stress	8.75 ± 8.37	15.30 ± 9.90	<0.001

NIU, Normal Internet use (YDQ score ≤ 4 points); PIU, Problematic Internet use (YDQ score ≥ 5 points).

### Interaction analysis of sex and IA

**Figure 1:**
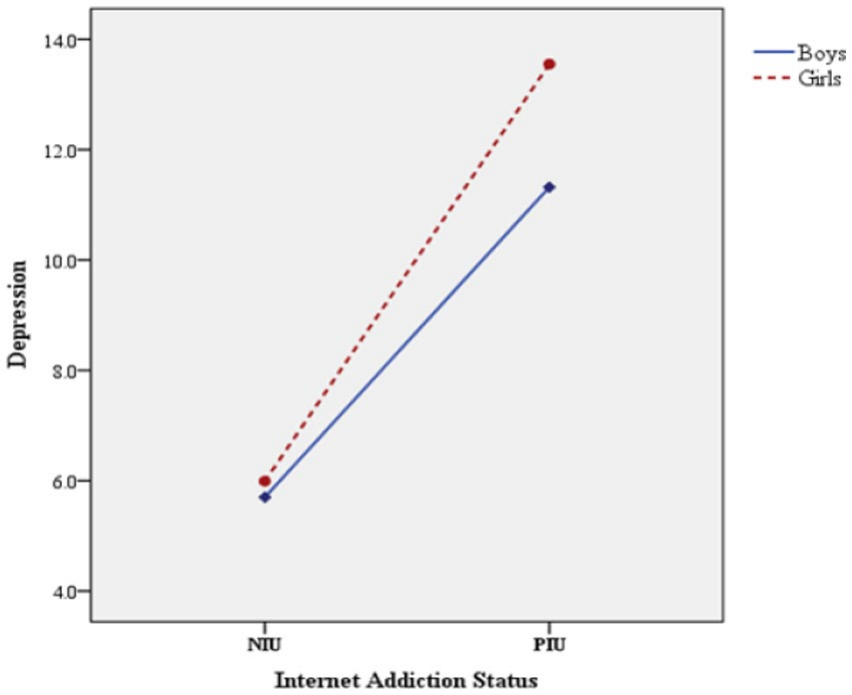
The interaction effect between sex and IA on depression.

**Figure 2:**
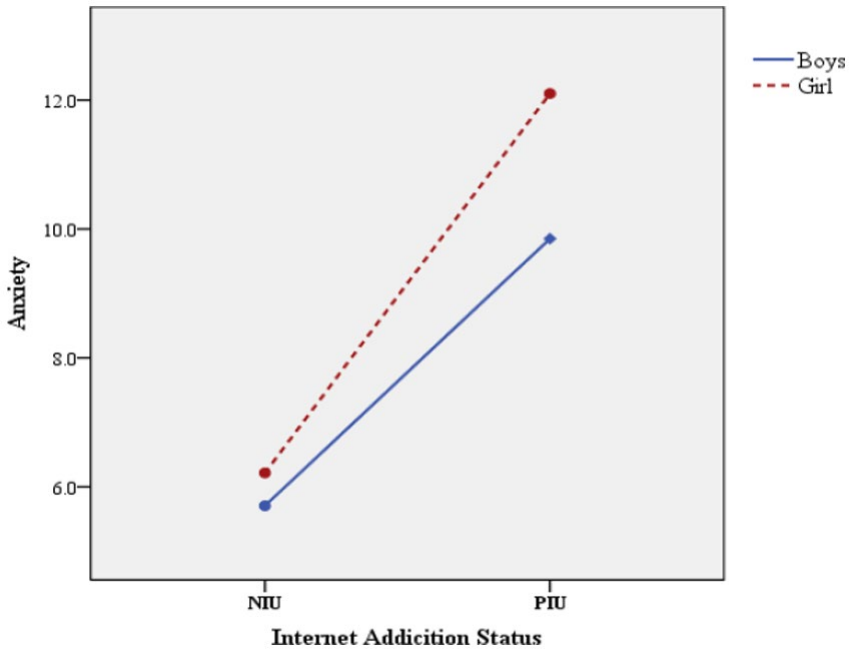
The interaction effect between sex and IA on anxiety.

**Figure 3:**
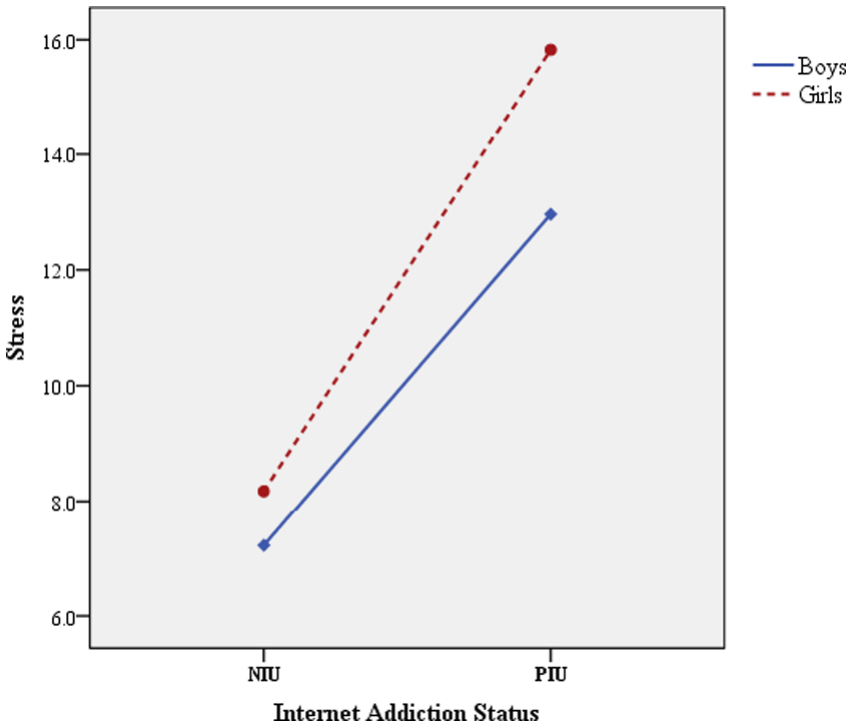
The interaction effect between sex and IA on stress.

The interaction between sex and IA was verified by two-way ANOVA (Table 4). The results showed that the main effects of sex and IA on depression, anxiety, and stress were significant (*p* < 0.05), and the interaction between sex and IA was also significant (*p* < 0.05). Figure 1 shows this interaction in the symptoms of depression. Figure 2 also shows the significant interaction between sex and IA in anxiety symptoms. Figure 3 shows the significant interaction between sex and IA in stress symptoms.

**Table 4:** Interaction analysis of sex and IA.

	df	*F*	*p*
Depression			
Sex	1	454.489	<0.001
IA	1	12410.418	<0.001
Sex*IA	1	269.215	<0.001
Anxiety			
Sex	1	630.952	<0.001
IA	1	8311.859	<0.001
Sex*IA	1	250.830	<0.001
Stress			
Sex	1	970.961	<0.001
IA	1	11945.789	<0.001
Sex*IA	1	243.197	<0.001

On the premise of significant interaction, simple effect analysis was conducted (Table 5). That is to fix one level of one factor and verify the difference of adolescents’ psychological problem scores among the levels of another factor. The results showed that there were significant differences in the scores of depression, anxiety and stress among boys in different Internet addiction states (*p* < 0.05). Specifically, the scores of depression, anxiety and stress of boys in PIU group were 5.619 points, 4.144 points and 5.741 points higher than those in NIU group, respectively. Similarly, this difference exists among girls, the scores of depression, anxiety and stress of girls in PIU group were 7.560 points, 5.886 points and 7.652 points higher than those in NIU group, respectively. The mean difference (PIU-NIU) of depression, anxiety and stress scores of girls in different Internet addiction groups were greater than that of boys (depression: 7.560 > 5.619, anxiety: 5.886 > 4.144, stress: 7.652 > 5.741). In addition, when the fixed Internet addiction status was in the NIU or PIU group, the scores of depression, stress and anxiety of adolescents of different sex were also different.

**Table 5:** Simple effect analysis of the interaction between sex and IA on psychological problems among adolescents.

Variables	df	Mean difference (95%CI)	*F*	*p*
Depression				
IA WITHIN Boys	1	5.619^a^ (5.458, 5.779)	4700.116	<0.001
IA WITHIN Girls	1	7.560^a^ (7.392, 7.727)	7853.287	<0.001
Sex WITHIN NIU	1	0.290^b^ (0.197, 0.384)	36.959	<0.001
Sex WITHIN PIU	1	2.231^b^ (2.019, 2.444)	425.186	<0.001
Anxiety				
IA WITHIN Boys	1	4.144^a^ (3.996, 4.293)	2955.765	<0.001
IA WITHIN Girls	1	5.886^a^ (5.731, 6.042)	5504.876	<0.001
Sex WITHIN NIU	1	0.511^b^ (0.423, 0.598)	132.005	<0.001
Sex WITHIN PIU	1	2.253^b^ (2.056, 2.450)	501.105	<0.001
Stress				
IA WITHIN Boys	1	5.741^a^ (5.574, 5.907)	4573.109	<0.001
IA WITHIN Girls	1	7.652^a^ (7.478, 7.825)	7498.755	<0.001
Sex WITHIN NIU	1	0.954^b^ (0.857, 1.051)	371.290	<0.001
Sex WITHIN PIU	1	2.864^b^ (2.645, 3.084)	653.043	<0.001

^a^Mean difference, score of psychological problems in PIU group – score of psychological problems in NIU group.

^b^Mean difference, girls psychological problem score – boys psychological problem score.

### Interaction analysis of grade and IA

**Figure 4:**
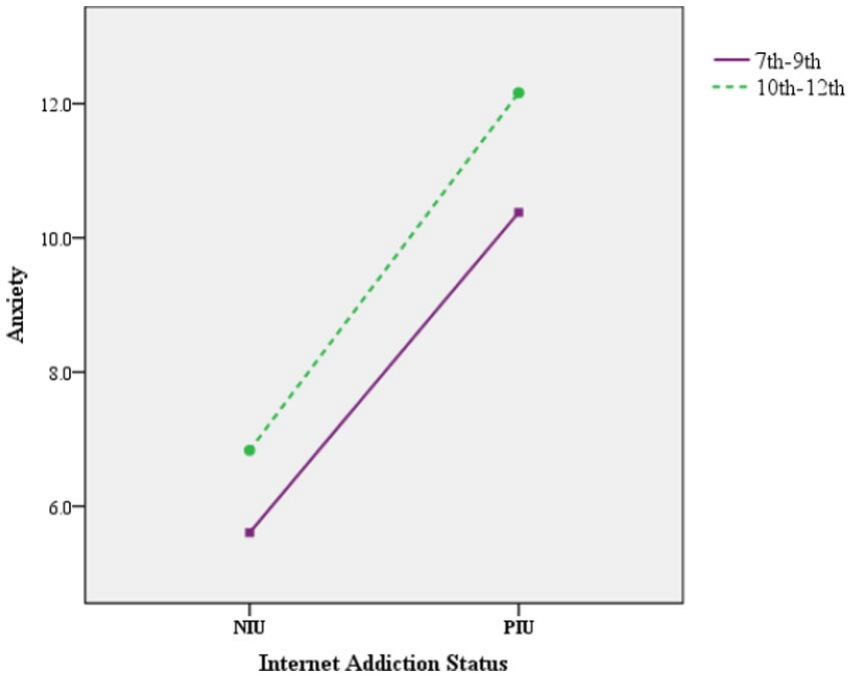
The interaction effect between grade and IA on anxiety.

However, for the grade, when grade was roughly divided into junior middle school (7th-9th) and senior high school (10th-12th), the main effects of grade on depression, anxiety and stress were significant (*p* < 0.05), but the interaction between grade and IA was only significant in the dimension of anxiety (*P*_Anx_ < 0.05, *P*_Dep_ = 0.461, *P*_Str_ = 0.752). Figure 4 shows the significant interaction between grade and IA in anxiety symptoms (Table 6).

**Table 6:** Interaction analysis of grade and IA.

	df	*F*	*p*
Depression			
Grade	1	320.043	<0.001
IA	1	10151.363	<0.001
Grade*IA	1	0.543	0.461
Anxiety			
Grade	1	629.814	<0.001
IA	1	7091.817	<0.001
Grade*IA	1	21.522	<0.001
Stress			
Grade	1	461.011	<0.001
IA	1	9679.666	<0.001
Grade*IA	1	0.100	0.752

The results of simple effect analysis showed that, there were significant differences in the score of anxiety among junior middle school students (7th-9th) in different Internet addiction states (*p* < 0.05). The score of anxiety of junior middle school students in PIU group were 4.771 points higher than those in NIU group. For high school students (grade 10th-12th), the difference was 5.328. Similarly, when the fixed Internet addiction status was in the NIU or PIU group, the score of anxiety of adolescents of different grade were also different (Table 7).

**Table 7:** Simple effect analysis of the interaction between grade and IA on anxiety among adolescents.

Variables	df	Mean difference (95%CI)	*F*	*p*
Anxiety				
IA WITHIN Grade 7–9	1	4.771^a^ (4.643, 4.900)	5306.789	<0.001
IA WITHIN Grade 10–12	1	5.328^a^ (5.131, 5.525)	2812.689	<0.001
Grade WITHIN NIU	1	1.227^b^ (1.132, 1.322)	641.891	<0.001
Grade WITHIN PIU	1	1.783^b^ (1.568, 1.998)	264.091	<0.001

^a^Mean difference, anxiety score of PIU group–anxiety score of NIU group.

^b^Mean difference, grade 10–12 anxiety score–grade 7–9 anxiety score.

In order to further understand the differences between the six grades of junior high school and senior high school, this study analyzes the grade as a six categories variable. Different from the results of grade as a binary variable, when grade was subdivided, the interaction between grade and IA was significant in depression, anxiety and stress (*p* < 0.05; Appendix Table S1; Appendix Figures S1–S3). The results of simple effect analysis showed that when the grade was fixed, the scores of depression, anxiety and stress of adolescents with different Internet addiction group were significantly different (Appendix Table S2). Among them, the difference of depression and stress scores of 8th grade students in different Internet addiction groups was the largest (mean difference was 6.862 and 6.904 respectively, *p* < 0.05). The anxiety score difference of grade 11 students was the largest (mean difference: 6.904, *p* < 0.05).

## Discussion

Adolescence is an important transition period for individuals from childish to mature, and their psychological and behavioral development is easily affected by the external environment. With the popularity of Internet and the spread of electronic games, adolescents are becoming the main use group (Bonfiglio et al., 2020). However, what appears at the same time is the problem of adolescents’ IA. Given the possible adverse consequences of IA, this study aims to explore the relationship between IA and psychological problems among adolescents.

### Status of Internet addiction among adolescents

At present, the prevalence of PIU has been reported in various countries and different populations (Kitazawa et al., 2018). The results of this study showed that the prevalence rate of PIU among adolescents (mean age: 14.76 ± 1.44) in Guangdong province was 16.32%, which seems to be slightly different from the results of others. The prevalence estimates of PIU among adolescents and young adults have been observed worldwide: results showed that PIU was about 1.2 to 8.2% in Europe (Bélanger et al., 2011; Villella et al., 2011; Poli and Agrimi, 2012), 1.0 to 12.0% in Middle East (Ak et al., 2013; Spada, 2014). And studies among Chinese adolescents (aged 12–20 years) also found that the prevalence of PIU was 5.5 to 17.2% (An et al., 2014; Mei et al., 2016; Tan et al., 2016). Thus, we may be able to say that the prevalence of PIU in this survey is higher than that in Europe and the Middle East, but it is similar to some studies results in China. The reasons for the above results may be due to the different sample size, economic development level and survey time. For example, during the coronavirus disease (COVID-19) pandemic, the traditional off-line teaching has been changed to online teaching, which may increase the chances youngsters to use the Internet. In view of the possible adverse consequences of Internet addiction, it is necessary to find effective preventive measures as soon as possible.

### Status of psychological problems among adolescents

This study also assessed the mental health problems of adolescents. In order to slow down the spread of the infection (COVID-19), most countries generally adopt measures of home quarantines and national school closer to maintain social distance. However, Wang et al. pointed out that school closure may cause disruptions in the social interaction, and mental health of children and adolescents (Wang et al., 2020). This survey found that the prevalence of depression, anxiety, and stress symptoms came in at 30.16, 35.97, and 18.80%, respectively, according to the adolescents’ scores on DASS-21, which was higher than Tang’s study on the psychological problems of primary and secondary school students in Shanghai (depression: 19.7%, anxiety: 24.9%, stress: 15.2%; Tang et al., 2021). This may be related to the different grades of the subjects investigated. As is known to all, secondary school students face more academic pressure than primary school students as they need to prepare for the Zhongkao (entrance examination for senior secondary school) and gaokao (the national higher education entrance examination). Besides, the new forms of learning, that is, studying alone at home without face-to-face interaction with teachers and classmates, may also increase the risk of psychological problems of middle school students.

### Comparative analysis of Internet addiction and psychological problems among adolescents of different sexes and grades

### Sex

Consistent with previous studies (Johansson and Götestam, 2004; Ko et al., 2005), this study found that the prevalence of PIU among boys (17.89%) was higher than that of girls (14.86%). This is related to the fact that boys are more likely to indulge in online games. Durkee et al. (2012) found that playing online games was the predominant activity for males, with nearly two-fold increases between adaptive and pathological use. Besides, boys tend to be unwilling to communicate with others and seek help (Shao et al., 2018). When facing life stress or negative life events, they are more willing to indulge in the virtual world to vent their emotions. It is worth noting that the issue seems to have been exacerbated by COVID-19-related lockdown restrictions, which turned online interaction into the only viable option for many (Elhai et al., 2020; Alheneidi et al., 2021). The above factors may contribute to a higher detection rate of PIU in boys. Yet, the psychological problems of girls were more serious than those of boys. The prevalence of depression, anxiety and stress in girls (30.74, 37.82, 20.89%) were significantly higher than that in boys (29.54, 33.99, 16.56%), which was consistent with previous research. Tolman et al. applied a feminist psychodynamic developmental framework to argue that the process of internalizing beliefs and behaviors about female roles and responsibilities exerts a deleterious effect on the psychological well-being of adolescent females (Tolman et al., 2006). Girls are often taught to build a considerable, passive, and graceful self-image since childhood (Mckenna et al., 2002). After entering puberty, the further strengthening of gender awareness will often limit the autonomy of girls’ behavior and suppress personality development (Samuels et al., 2017), which increases the risk of psychological problems. However, for boys, as mentioned above, they are often easy to release bad emotion through social activities, such as online games and physical exercises. Similarly, the impact of the COVID-19 pandemic on girl’s negative emotions cannot be ignored. Due to preventative measures—e.g., quarantine, lockdown, and social distancing—students have to stay at home and study online (Unger and Meiran, 2020). When the conventional offline learning mode is changed to online learning, some students may fall into anxiety due to the change of learning mode. There were studies found that during the lockdowns of the school around the globe, academic performance could be best the correlate of anxiety, especially among female students (Al-Dwaikat et al., 2020; Cannito et al., 2022).

### Grade

This study also found that there were differences in IA and psychological problems among adolescents in different grades. When grades were roughly divided into junior middle school (7th-9th) and senior high school (10th-12th), there was no significant difference in the prevalence of PIU between different grades (*p* = 0.793). However, when grades were divided into six grades, there were significant differences in the prevalence of PIU among adolescents (*p* < 0.05). In the stage of junior middle school (7th-9th), with the growth of grade, the prevalence of PIU in adolescents increases gradually (15.61 < 16.57 < 17.29%). But in high school (10th-12th), the prevalence of PIU decreased. This result confirms the views of Widyanto and Mcmurran. The gradual decrease for the prevalence of PIU might be due to increased cognitive maturation and engagement in meaningful activities (Shek and Yu, 2016). According to the cognitive-behavioral model proposed by Davis, individual’s cognitions (or thoughts) as the main source of abnormal behavior (such as PIU) (Davis, 2001). When there are distortions in both self-cognition and external cognition, individuals often have cognitive obstacles in some aspects. For example, believe in the network environment rather than the real world, resulting in self-doubt. The cognitive impairment of individuals with addictive tendencies in these aspects will aggravate the symptoms of individual Internet addiction (Mai et al., 2012). With the deepening of individual’s familiarity with the Internet, as well as the growth of age, and the aggravation of learning tasks, this may lead to changes in adolescents’ original cognition of the Internet.

At the same time, there were significant differences in psychological problems among adolescents in different grades. In general, the prevalence of depression, anxiety and stress in senior high school students (35.27, 42.32, 21.87%) was higher than that in junior high school students (27.99, 33.26, 17.49%). Specifically, the prevalence of depression, anxiety and stress of grade 11 students were the highest, followed by grade 12. This is largely due to the different learning pressures in different grades (Ang and Huan, 2006). In China, the curriculum of junior middle school is relatively easy, but the curriculum of senior high school is more difficult. Moreover, adolescents will face the problem of curriculum selection in grade 11, so changes in the environment may lead to emotional instability. In addition, with the growth of age, the expansion of social contact and the enhancement of self-awareness, senior students’ thinking is more delicate and introverted, and the ideological problems they face are increasing.

### Interaction between sex and Internet addiction

In view of the differences in the scores of adolescents’ psychological problems under different Internet addiction states (Table 3), this study further analyzes the impact of the interaction between gender and IA on psychology. The results showed that girls in PIU group had the highest scores of depression, anxiety and stress. There are several reasons for the great impact of Internet addiction on depression, anxiety, and stress symptoms in girls. Ordinarily, girls tend to use the Internet to blog, chat, update their home pages, send messages and search information (Heo et al., 2014), but some girls may play games and make boyfriends online. Meanwhile, girls often receive more family supervision than boys, which may prevent girls from spending as much time on online gaming (Ko et al., 2005). In this case, it is bound to affect the relationship between girls and their families. Second, because they are in adolescence, both boys and girls will face pressure from learning, peers, parents and others. However, featured with sensitive perception and delicate sentiment, girls are more likely to be influenced by external factors. For them, avoidance of real life may lead to harsh self-criticism, which aggravates their negative feelings (Guo et al., 2020). Therefore, girls in PIU group have a higher risk of psychological problems.

### Interaction between grade and Internet addiction

When grade was roughly divided into junior middle school (7th-9th) and senior high school (10th-12th), the interaction between grade and IA was only significant in the psychological problem of anxiety. But when grades were divided into six grades, the interaction between grade and IA has statistical significance in the three dimensions of psychological problems. Specifically, although the prevalence of PIU was the highest in grade 9, the influence of IA on depression and stress was more obvious in grade 8 and grade 9 students, and the influence of IA on anxiety was more obvious in grade 11 students. Middle school students are in a special stage of physical and mental development. Their self-consciousness is gradually awakening, and the contradictions and psychological conflicts they face are also gradually increasing. The Internet is the most convenient means for them to express themselves. However, once addicted to the Internet and trapped in the virtual world, middle school students will be separated from life and society, lose interest in learning, and easily fall into depression. This phenomenon is more significant among grade 8 and 9 students because for grade 7 students, they have just integrated into the new environment, so they need an adaptation process. At the same time, with the growth of grade, students’ pressure is also increasing. This pressure is largely due to the mismatch between parents’ expectations and adolescents’ own abilities. The reason why high school students have less pressure is that their physical and mental development is relatively mature and they have a certain ability to deal with pressure. For grade 11 students, the learning task at this stage is heavier. On the one hand, students need a process of adaptation after course selection. On the other hand, in order to buy more review time, the curriculum arrangement of some schools is relatively compact. When the temptation of the Internet and heavy learning tasks cannot be balanced, adolescents are likely to fall into anxiety.

## Strengths and limitations

This study has several strengths: firstly, different from previous studies, this study further analyzes the relationship between Internet addiction and psychological problems in different populations (analysis of sex and grade heterogeneity). For example, most previous studies indicated that boy was a risk factor of Internet addiction. But this study further verifies that although the prevalence of PIU was higher in boys, the adverse effect of Internet addiction was more obvious in girls. Secondly, this study is based on a large sample cross-sectional survey in Guangdong Province. Therefore, the results of this study can provide guidance for relevant government departments to formulate adolescent health management and health promotion plans.

Several limitations in the present study should be considered. First, this survey was conducted during the COVID-19 pandemic. Li et al. found the prevalence of Internet addiction among the general population in China during the COVID-19 pandemic was remarkably high (Li et al., 2021). This may be related to measures to encourage people to stay at home for maintaining social distancing in order to curb the development of the epidemic (Ortiz de Gortari, 2022). The measure of isolation at home reduces the opportunities for people to go out for activities, while the free time of Internet access at home increases (Kar et al., 2020). Therefore, this may be one of the reasons for the high rate of Internet addiction among adolescents in this survey. Second, because this study is a cross-sectional survey of a large sample, considering the response rate of subjects and the effectiveness of the questionnaire, the design of the research content is relatively limited. In the future research, other contents (such as cognitive factors) can be added for more in-depth discussion. Third, the cross-sectional design in the present study could not allow inferring cause–effect relationship between IA and psychological problems, longitudinal studies are needed to explore the detailed causal relationships. And since this study is an online survey and self-reporting was adopted, so self-bias may have an influence on the accuracy of the results. More measurement methods should be considered for referencing in future research. Despite these limitations, our findings still have important reference value in formulating targeted interventions.

## Conclusion

Adolescents often have strong curiosity and are willing to pursue more autonomy. This makes it particularly important to guide them to cultivate healthy behavior. The present study revealed that IA was prevalent among adolescents in Guangdong province and the prevalence of PIU was 16.32%. In addition, psychological problems such as depression (30.16%), anxiety (35.97%) and stress (18.80%) also cannot be ignored. Although the prevalence of PIU was higher in boys, girls in PIU group had the highest scores of depression, anxiety and stress. Similarly, the influence of IA on depression and stress was more obvious in grade 8 and grade 9 students, and the influence of IA on anxiety was more obvious in grade 11 students. The results suggest that we should not only consider the high prevalence of boys and grade 9 students, but also explore the possible negative effects of Internet addiction on the characteristics of different groups. Thus, we recommend integrating long-term follow-ups into the associated mental health programs of adolescents to monitor the development of Internet addiction and psychological problems. Meanwhile, scholars and health practitioners should consider gender and grade differences when formulating strategies to prevent IA, so that it could offer significative guidance for clinicians, parents and educators.

## Data availability statement

The raw data supporting the conclusions of this article will be made available by the authors, without undue reservation.

## Ethics statement

Ethical approval for this study was obtained from Southern Medical University. Written informed consent to participate in this study was provided by the participants’ legal guardian/next of kin. Written informed consent was obtained from the individual(s), and minor(s)’ legal guardian/next of kin, for the publication of any potentially identifiable images or data included in this article.

## Author contributions

CZ designed this study, participated in its implementation, and served as the lead writer. YX and BX did the data interpretation and co-wrote the article. XZ, LS, and PL were involved in the study design and critically revised the article. SX, FD, and JZ helped to collect the data and research the literature. YC, YL, and ZQ helped with formatting of this manuscript. All authors have seen and approved the final manuscript.

## Funding

This study was funded by the Education Science Planning Project of Guangdong Province in 2022 (Grant number: 2022JKZG048), Special Research Project of Prevention and Control during the COVID-19 pandemic in Universities of Guangdong (Grant number: 2020KZDZX1046); and Key Laboratory of Philosophy and Social Sciences of Guangdong Higher Education Institutions for Health Polices Research and Evaluation (Grant number: 2015WSY0010).

## Conflict of interest

The authors declare that the research was conducted in the absence of any commercial or financial relationships that could be construed as a potential conflict of interest.

## Publisher’s note

All claims expressed in this article are solely those of the authors and do not necessarily represent those of their affiliated organizations, or those of the publisher, the editors and the reviewers. Any product that may be evaluated in this article, or claim that may be made by its manufacturer, is not guaranteed or endorsed by the publisher.
