# Supplementary material for: Associations between internet addiction and psychological problems among adolescents: description and possible explanations
Source: Front Psychol. 2023 May 15;14:1097331. doi: 10.3389/fpsyg.2023.1097331 (PMC10226521; doi:10.3389/fpsyg.2023.1097331)
Supplement: Supplementary file 1 [file Data_Sheet_1.docx]

**Supplemental Materials**

Table S1 Interaction analysis of grade and Internet addiction

|  | *df* | *F* | *P* |
| --- | --- | --- | --- |
| Depression | | |  |
| Grade | 5 | 90.170 | <0.001 |
| IA | 1 | 7114.919 | <0.001 |
| Grade*IA | 5 | 4.713 | <0.001 |
| Anxiety | | |  |
| Grade | 5 | 159.670 | <0.001 |
| IA | 1 | 4985.294 | <0.001 |
| Grade*IA | 5 | 12.889 | <0.001 |
| Stress | | |  |
| Grade | 5 | 136.963 | <0.001 |
| IA | 1 | 6772.834 | <0.001 |
| Grade*IA | 5 | 4.903 | <0.001 |


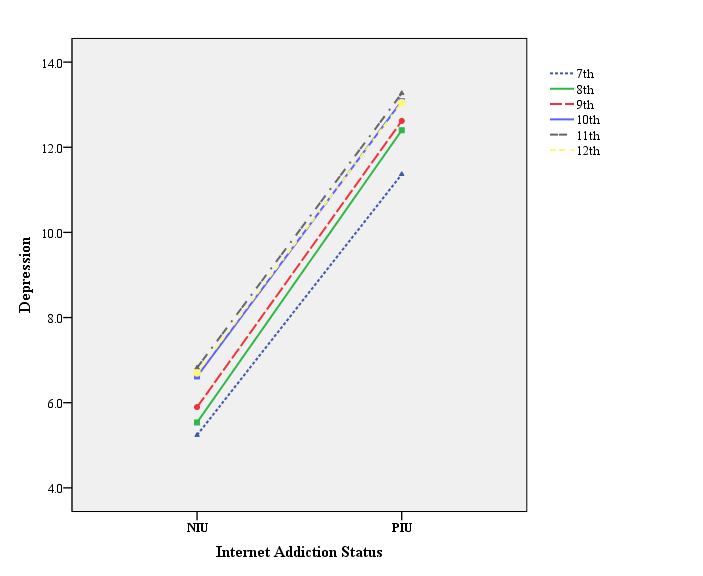


Figure S1 The interaction effect between grade (7th-12th) and Internet addiction on depression


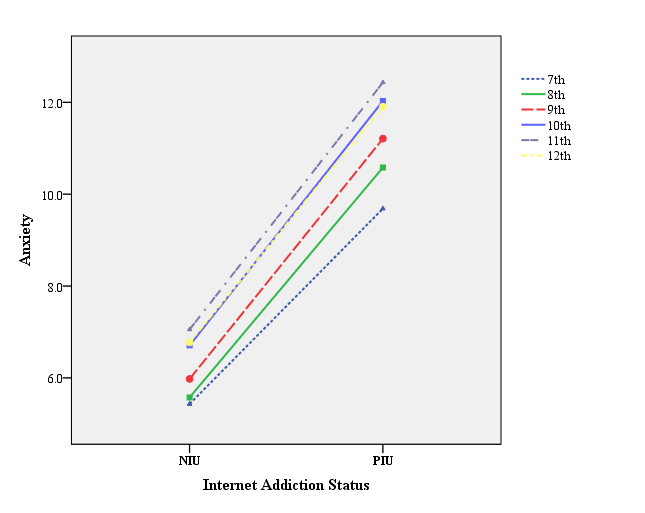


Figure S2 The interaction effect between grade (7th-12th) and Internet addiction on anxiety


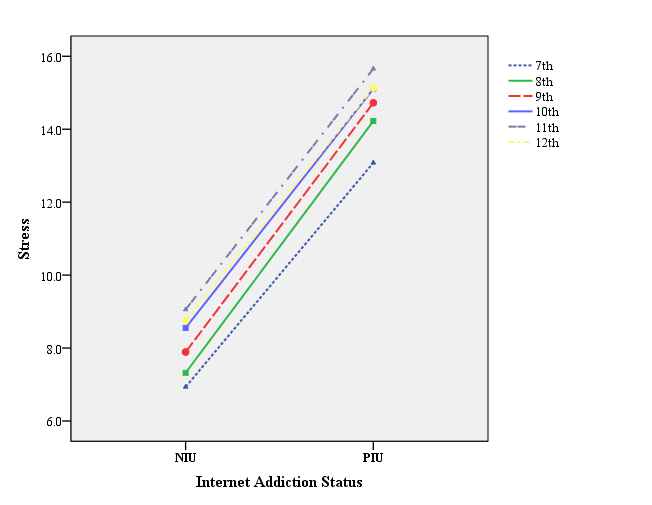


Figure S3 The interaction effect between grade (7th-12th) and Internet addiction on stress

Table S2 Simple effect analysis of the interaction between grade and Internet addiction on psychological problems among adolescents

| Variables | *df* | Mean difference (*95%CI*) | *F* | *P* |
| --- | --- | --- | --- | --- |
| Depression |  |  |  |  |
| IA WITHIN grade 7th | 1 | 6.125^a^ (5.907, 6.342) | 3045.935 | <0.001 |
| IA WITHIN grade 8th | 1 | **6.862^a^** (6.631, 7.094) | 3367.925 | <0.001 |
| IA WITHIN grade 9th | 1 | 6.722^a^ (6.442, 7.003) | 2209.677 | <0.001 |
| IA WITHIN grade 10th | 1 | 6.468^a^ (6.180, 6.755) | 1943.483 | <0.001 |
| IA WITHIN grade 11th | 1 | 6.446^a^ (6.090, 6.801) | 1260.997 | <0.001 |
| IA WITHIN grade 12th | 1 | 6.351^a^ (5.694, 7.008) | 359.276 | <0.001 |
| Grade WITHIN NIU | 5 | - | 123.537 | <0.001 |
| Grade WITHIN PIU | 5 | - | 32.717 | <0.001 |
| Anxiety |  |  |  |  |
| IA WITHIN grade 7th | 1 | 4.253^a^ (4.051, 4.455) | 1699.804 | <0.001 |
| IA WITHIN grade 8th | 1 | 5.007^a^ (4.792, 5.223) | 2075.224 | <0.001 |
| IA WITHIN grade 9th | 1 | 5.233^a^ (4.973, 5.494) | 1549.916 | <0.001 |
| IA WITHIN grade 10th | 1 | 5.322^a^ (5.054, 5.589) | 1522.644 | <0.001 |
| IA WITHIN grade 11th | 1 | **5.377**^a^ (5.046, 5.707) | 1015.438 | <0.001 |
| IA WITHIN grade 12th | 1 | 5.134^a^ (4.523, 5.744) | 271.670 | <0.001 |
| Grade WITHIN NIU | 5 | - | 144.390 | <0.001 |
| Grade WITHIN PIU | 5 | - | 74.854 | <0.001 |
| Stress |  |  |  |  |
| IA WITHIN grade 7th | 1 | 6.146^a^ (5.920, 6.371) | 2854.614 | <0.001 |
| IA WITHIN grade 8th | 1 | **6.904**^a^ (6.664, 7.144) | 3173.221 | <0.001 |
| IA WITHIN grade 9th | 1 | 6.832^a^ (6.542,7.123) | 2124.765 | <0.001 |
| IA WITHIN grade 10th | 1 | 6.554^a^ (6.256, 6.852) | 1857.331 | <0.001 |
| IA WITHIN grade 11th | 1 | 6.599^a^ (6.231, 6.968) | 1230.379 | <0.001 |
| IA WITHIN grade 12th | 1 | 6.378^a^ (5.697, 7.059) | 337.273 | <0.001 |
| Grade WITHIN NIU | 5 | - | 182.510 | <0.001 |
| Grade WITHIN PIU | 5 | - | 49.283 | <0.001 |

a: Mean difference, score of psychological problems in PIU group – score of psychological problems in NIU group.

Table S3 The results of post hoc test (dependent variable: depression)

| Variables |  |  | Mean difference (*95%CI*) | *P* |
| --- | --- | --- | --- | --- |
| NIU | Grade 7th | 8th | -0.296 (-0.424, -0.169) | <0.001 |
|  |  | 9th | -0.657^a^ (-0.802, -0.512) | <0.001 |
|  |  | 10th | -1.377^a^ (-1.521, -1.233) | <0.001 |
|  |  | 11th | -1.582^a^ (-1.752, -1.412) | <0.001 |
|  |  | 12th | -1.459^a^ (-1.729, -1.188) | <0.001 |
|  |  |  |  |  |
|  | Grade 8th | 9th | -0.361^a^ (-0.511, -0.211) | <0.001 |
|  |  | 10th | -1.081^a^ (-1.230, -0.932) | <0.001 |
|  |  | 11th | -1.285^a^ (-1.460, -1.111) | <0.001 |
|  |  | 12th | -1.162^a^ (-1.436, -0.889) | <0.001 |
|  |  |  |  |  |
|  | Grade 9th | 10th | -0.720^a^ (-0.884, -0.556) | <0.001 |
|  |  | 11th | **-0.925**^a^ (-1.112, -0.737) | <0.001 |
|  |  | 12th | -0.802^a^ (-1.083, -0.520) | <0.001 |
|  |  |  |  |  |
|  | Grade 10th | 11th | -0.205 (-0.391, -0.018) | 0.031 |
|  |  | 12th | -0.082 (-0.363, 0.199) | 0.568 |
|  |  |  |  |  |
|  | Grade 11th | 12th | 0.123 (-0.172, 0.418) | 0.414 |
|  |  |  |  |  |
| PIU | Grade 7th | 8th | -1.034 (-1.325, -0.743) | <0.001 |
|  |  | 9th | -1.255 (-1.579, -0.931) | <0.001 |
|  |  | 10th | -1.720 (-2.051, -1.390) | <0.001 |
|  |  | 11th | -1.903 (-2.284, -1.522) | <0.001 |
|  |  | 12th | -1.685 (-2.322, -.1.048) | <0.001 |
|  |  |  |  |  |
|  | Grade 8th | 9th | -0.221 (-0.552, 0.110) | 0.191 |
|  |  | 10th | -0.686 (-1.024, -0.348) | <0.001 |
|  |  | 11th | -.869 (-1.256, -0.482) | <0.001 |
|  |  | 12th | -0.651 (-1.292, -0.011) | 0.046 |
|  |  |  |  |  |
|  | Grade 9th | 10th | -0.465 (-0.832, -0.099) | 0.013 |
|  |  | 11th | -0.648 (-1.060, -0.236) | 0.002 |
|  |  | 12th | -0.430 (-1.086, 0.226) | 0.199 |
|  |  |  |  |  |
|  | Grade 10th | 11th | -0.183 (-0.600, 0.235) | 0.392 |
|  |  | 12th | 0.035 (-0.624, 0.695) | 0.917 |
|  |  |  |  |  |
|  | Grade 11th | 12th | 0.218 (-0.468, 0.905) | 0.534 |

Table S4 The results of post hoc test (dependent variable: Anxiety)

| Variables |  |  | Mean difference (*95%CI*) | *P* |
| --- | --- | --- | --- | --- |
| NIU | Grade 7th | 8th | -0.141 (-0.260, -0.023) | 0.020 |
|  |  | 9th | -0.544 (-0.679, -0.410) | <0.001 |
|  |  | 10th | -1.276 (-1.410, -1.142) | <0.001 |
|  |  | 11th | -1.623 (-1.781, -1.465) | <0.001 |
|  |  | 12th | -1.340 (-1.591, -1.089) | <0.001 |
|  |  |  |  |  |
|  | Grade 8th | 9th | -0.403 (-0.542, -0.264) | <0.001 |
|  |  | 10th | -1.135 (-1.273, -0.996) | <0.001 |
|  |  | 11th | -1.481 (-1.643, -1.319) | <0.001 |
|  |  | 12th | -1.199 (-1.453, -0.945) | <0.001 |
|  |  |  |  |  |
|  | Grade 9th | 10th | -0.732 (-0.884, -0.580) | <0.001 |
|  |  | 11th | -1.078 (-1.252, -0.904) | <0.001 |
|  |  | 12th | -0.796 (-1.058, -0.534) | <0.001 |
|  |  |  |  |  |
|  | Grade 10th | 11th | -0.346 (-0.520, -0.173) | <0.001 |
|  |  | 12th | -0.064 (-0.325, 0.197) | 0.632 |
|  |  |  |  |  |
|  | Grade 11th | 12th | 0.283 (0.008, 0.557) | 0.044 |
|  |  |  |  |  |
| PIU | Grade 7th | 8th | -0.895 (-1.166, -0.625) | <0.001 |
|  |  | 9th | -1.525 (-1.826, -1.223) | <0.001 |
|  |  | 10th | -2.345 (-2.652, -2.037) | <0.001 |
|  |  | 11th | -2.746 (-3.100, -2.392) | <0.001 |
|  |  | 12th | -2.221 (-2.812, -1.629) | <0.001 |
|  |  |  |  |  |
|  | Grade 8th | 9th | -0.629 (-0.937, -0.321) | <0.001 |
|  |  | 10th | -1.449 (-1.763, -1.135) | <0.001 |
|  |  | 11th | -1.851 (-2.211, -1.491) | <0.001 |
|  |  | 12th | -1.325 (-1.921, -0.730) | <0.001 |
|  |  |  |  |  |
|  | Grade 9th | 10th | -0.820 (-1.161, -0.479) | <0.001 |
|  |  | 11th | -1.222 (-1.605, -0.838) | <0.001 |
|  |  | 12th | -0.696 (-1.306, -0.086) | 0.025 |
|  |  |  |  |  |
|  | Grade 10th | 11th | -0.401 (-0.790, -0.013) | 0.043 |
|  |  | 12th | 0.124 (-0.489, 0.737) | 0.691 |
|  |  |  |  |  |
|  | Grade 11th | 12th | 0.526 (-0.112, 1.163) | 0.106 |

Table S5 The results of post hoc test (dependent variable: Stress)

| Variables |  |  | Mean difference (*95%CI*) | *P* |
| --- | --- | --- | --- | --- |
| NIU | Grade 7th | 8th | -0.386 (-0.518, -0.253) | <0.001 |
|  |  | 9th | -0.957 (-1.107, -0.807) | <0.001 |
|  |  | 10th | -1.618 (-1.767, -1.469) | <0.001 |
|  |  | 11th | -2.123 (-2.299, -1.947) | <0.001 |
|  |  | 12th | -1.817 (-2.097, -1.537) | <0.001 |
|  |  |  |  |  |
|  | Grade 8th | 9th | -0.571 (-0.726, -0.416) | <0.001 |
|  |  | 10th | -1.232 (-1.386, -1.078) | <0.001 |
|  |  | 11th | -1.737 (-1.918, -1.556) | <0.001 |
|  |  | 12th | -1.431 (-1.714, -1.148) | <0.001 |
|  |  |  |  |  |
|  | Grade 9th | 10th | -0.661 (-0.831, -0.491) | <0.001 |
|  |  | 11th | -1.166 (-1.360, -0.972) | <0.001 |
|  |  | 12th | -0.860 (-1.152, -0.568) | <0.001 |
|  |  |  |  |  |
|  | Grade 10th | 11th | -0.505 (-0.698, -0.312) | <0.001 |
|  |  | 12th | -0.199 (-0.490, 0.092) | 0.180 |
|  |  |  |  |  |
|  | Grade 11th | 12th | 0.306 (0.001, 0.612) | 0.050 |
|  |  |  |  |  |
| PIU | Grade 7th | 8th | -1.144 (-1.446, -0.843) | <0.001 |
|  |  | 9th | -1.644 (-1.979, -1.308) | <0.001 |
|  |  | 10th | -2.026 (-2.369, -1.683) | <0.001 |
|  |  | 11th | -2.577 (-2.971, -2.182) | <0.001 |
|  |  | 12th | -2.049 (-2.709, -1.389) | <0.001 |
|  |  |  |  |  |
|  | Grade 8th | 9th | -0.499 (-0.843, -0.156) | 0.004 |
|  |  | 10th | -0.882 (-1.232, -0.531) | <0.001 |
|  |  | 11th | -1.432 (-1.834, -1.031) | <0.001 |
|  |  | 12th | -0.905 (-1.569, -0.241) | 0.008 |
|  |  |  |  |  |
|  | Grade 9th | 10th | -0.382 (-0.762, -0.002) | 0.049 |
|  |  | 11th | -0.933 (-1.360, -0.506) | <0.001 |
|  |  | 12th | -0.406 (-1.086, 0.274) | 0.242 |
|  |  |  |  |  |
|  | Grade 10th | 11th | -0.551 (-0.984, -0.118) | 0.013 |
|  |  | 12th | -0.024 (-0.707, 0.660) | 0.946 |
|  |  |  |  |  |
|  | Grade 11th | 12th | 0.527 (-0.184, 1.238) | 0.146 |
